# TIMED-Design: flexible and accessible protein sequence design with convolutional neural networks

**DOI:** 10.1093/protein/gzae002

**Published:** 2024-01-30

**Authors:** Leonardo V Castorina, Suleyman Mert Ünal, Kartic Subr, Christopher W Wood

**Affiliations:** School of Informatics, University of Edinburgh, 10 Crichton Street, Edinburgh EH8 9AB United Kingdom; School of Biological Sciences, University of Edinburgh, Roger Land Building, Edinburgh EH9 3FF, United Kingdom; School of Informatics, University of Edinburgh, 10 Crichton Street, Edinburgh EH8 9AB United Kingdom; School of Biological Sciences, University of Edinburgh, Roger Land Building, Edinburgh EH9 3FF, United Kingdom

**Keywords:** protein sequence design, Convolutional Neural Networks (CNNs), user interface (UI), AlphaFold 2

## Abstract

Sequence design is a crucial step in the process of designing or engineering proteins. Traditionally, physics-based methods have been used to solve for optimal sequences, with the main disadvantages being that they are computationally intensive for the end user. Deep learning-based methods offer an attractive alternative, outperforming physics-based methods at a significantly lower computational cost. In this paper, we explore the application of Convolutional Neural Networks (CNNs) for sequence design. We describe the development and benchmarking of a range of networks, as well as reimplementations of previously described CNNs. We demonstrate the flexibility of representing proteins in a three-dimensional voxel grid by encoding additional design constraints into the input data. Finally, we describe TIMED-Design, a web application and command line tool for exploring and applying the models described in this paper. The user interface will be available at the URL: https://pragmaticproteindesign.bio.ed.ac.uk/timed. The source code for TIMED-Design is available at https://github.com/wells-wood-research/timed-design.

## Graphical Abstract

**Graphical Abstract:**
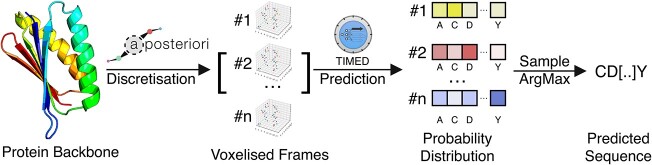

## Introduction

Protein design is a rapidly maturing field with an ever-increasing number of examples of designed proteins being produced (Woolfson, 2021). Excitingly, the field is moving beyond designing structures towards creating functional proteins (Pan & Kortemme, 2021), fulfilling a promise that has been repeated by protein designers for decades. Most protein design algorithms can be broken down into two phases: backbone design and sequence design. There are several approaches to backbone design, including fragment-based methods (Ferruz *et al.*, 2021, Krivacic *et al.*, 2022; Zhou *et al.*, 2020), parametric methods (Wood *et al.*, 2017; Yang *et al.*, 2021) and, more recently, deep learning (DL)-based methods (Watson *et al.*, 2023). Once backbone models have been generated, sequences must be selected that will fold to the target structure.

There are many approaches to sequence design too, including consensus design (Porebski & Buckle, 2016), Monte Carlo based sampling, such as the method employed by Rosetta (Leman *et al.*, 2020), and DL-based methods. DL-based methods offer several potential advantages over other methods: (1) while training DL models is computationally expensive, their application is usually much cheaper. This shifts the computational burden from the end user to the method developer, which improves accessibility of the method. (2) Given a rich enough data set, these methods can learn complex relationships that are present in the training data set without having to explicitly define these. For example, it is likely that the method will be biased by the training set to produce sequences that are more likely to be compatible with cellular environments. (3) As more training data become available, the performance of DL-based models increases without any change to the model architecture (Prapas *et al.*, 2021).

A range of neural network architectures have been explored for sequence design, including Convolutional Neural Networks (CNNs) (Anand *et al.*, 2022; Qi & Zhang, 2020; Zhang *et al.*, 2020), message-passing Graph Neural Networks (GNNs) (Dauparas *et al.*, 2022a) and Large Language Models (Ferruz *et al.*, 2022; Nijkamp *et al.*, 2022). CNNs have a range of useful properties that make them well suited to sequence design. CNNs are adept at learning spatial relationships and have been applied to many problems involving images (Deng *et al.*, 2009; Lin *et al.*, 2014). CNNs can be generalised beyond two dimensions, and can be applied to 3D data, where 3D voxels’ replace 2D pixels, enabling them to be applied to protein structure data.

**Fig. 1:**
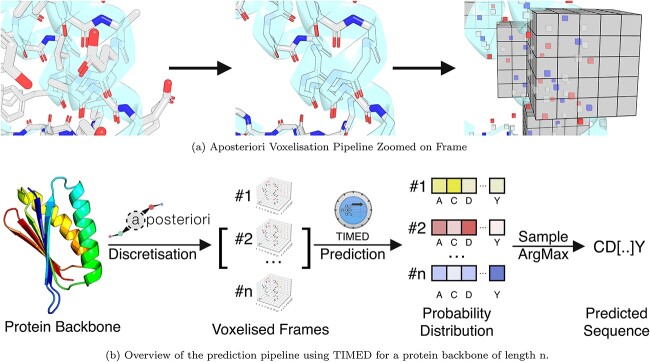
(**a**) Voxelisation pipeline from structures to frames. Protein sidechain atoms are removed from the structures to leave just the backbone atoms. Then, the backbone atoms are discretised into voxels. Finally, we extract a fixed cube of space around each amino acid that we call a ‘frame’. The frames contain atom voxels with the C$\alpha $ in its centre. (**b**) Aposteriori generates one frame for each amino acid in the backbone. For each frame, TIMED produces a probability distribution over the 20 amino acids. The output sequence can be obtained by either sampling from this probability distribution or by selecting the amino acid with the highest probability using the ArgMax function.

In order to perform sequence design with CNNs, the protein structure must be discretised into voxels (Fig. 1a), with each voxel containing information regarding its content, which is usually an identifier for an atom element or type. Regions around a particular residue are used as the input to the network, and a probability distribution for the identity of the amino acid is produced in the output. Training data sets can be generated using experimentally determined protein structures, with the aim of recovering the native sequence.

In this paper, we explore the application of CNNs to protein sequence design. We describe the development of TIMED, a CNN-based sequence design algorithm, as well as the reimplementation of a range of other CNN-based methods from the literature, that were not previously available. We explore the flexibility of the CNNs by encoding additional design constraints into the voxel data, enabling the designer to tune the properties of their designs. Finally, we described TIMED-Design, a web application (https://pragmaticproteindesign.bio.ed.ac.uk/timed) that enables the use of all of the CNN-based methods described in this paper. The source code and models described in this paper are open source and available on GitHub (https://github.com/wells-wood-research/timed-design).

## Methods

### Dataset generation

To begin training CNN models, we generated voxelised structures of proteins. To facilitate this process, we have developed an open-source Python library called Aposteriori (https://github.com/wells-wood-research/aposteriori), which offers features such as multi-processing, compression, and various types of atom encodings. For every amino acid in the input structure(s), we define a cubic region of space around it, with an edge length equal to --frame_edge_length (default to 12 Å). This region is then mapped to discrete space with a specified number of voxels per edge, denoted by --voxels-per-side, (default to 21 voxels). To ensure consistency, we rotate the protein structure so that the C$\alpha $ atom of the input amino acid positioned at the center of the frame with the N–C$\alpha $–C plane lying on the XY plane of the voxel grid. We create a frame for each residue in the protein sequence. Side chain information is removed; the C, N, O, C$\alpha $ atom and a virtual C$\beta $ position are voxelised within the frame, and are represented by a Gaussian function whose size depends on the van der Waals radius of the atom, as described in Zhang *et al.* (2020). The library generates a .hdf5 object, which can be manipulated as a Python dictionary. Additional technical details can be found in the code repository associated with this publication.

As shown in Fig. 1(b), CNN models are trained to classify frames, predicting probabilities for the twenty amino acid classes. Although frames have overlapping information, each frame is predicted independently of each other. Thus, for a protein of $n$ amino acids, the output is an array of shape (*n*, 20), containing the probability distributions for the identity of each amino acid. The probability distribution from CNN models allows us to explore the design space by weighted random sampling to generate new sequences. These sequences can then be folded using methods like AlphaFold (Jumper *et al.*, 2021) and screened using methods such as DE-STRESS (Stam & Wood, 2021).

### Training models with undersampling

The natural frequency of amino acid is not uniformly distributed, with some amino acids being more common than others. We use a random under sampling method to prevent CNN models from learning this frequency bias. Specifically, we cap the number of frames for each amino acid to match the count of cysteine, the least abundant amino acid in our training set. We perform random under sampling for the training and validation sets. At the beginning of each epoch, residues with a count higher than the minimum are randomly resampled from the discarded frames. This approach effectively increases the number of residues observed by the network while addressing the amino acid frequency bias.

The CNN models were built on the Keras framework (Abadi *et al.*, 2015). All of the models presented in this paper were trained using the same culled PDB data set from PISCES (cullpdb_pc90_res3.0_R1.0_d200702_chains40583) containing over 35K non-redundant protein structures (40K+ chains), with resolutions up to 3.0 Å (see Supplementary Section 3; Wang & Dunbrack, 2003).

### Models tested

We performed fixed-backbone sequence design with a range of methods, both physics- and DL-based:

#### Existing methods

- **EvoEF2 (Physics)**: uses several energy functions, including hydrogen bonding, electrostatic attractions and van der Waals interactions (Huang *et al.*, 2020).
- **Rosetta (Physics)**: uses Monte Carlo methods to optimise the sequence for a template structure (Ludwiczak *et al.*, 2018).
- **ProDCoNN (CNN)**: replicates the CNN architecture described in Zhang *et al.* (2020).
- **DenseNet (CNN)**: implements the DenseNet architecture for image classification proposed by Huang *et al.* (2019), but converted the 2D operations into 3D.
- **DenseCPD (CNN)**: implements Qi & Zhang (2020) proposed using a 3D DenseNet-inspired architecture for sequence design.
- **ProteinMPNN (GNN)**: a GNN method using a message passing architecture by Dauparas *et al.* (2022a).

#### Novel models

- **TIMED (CNN)**: stands for Three-dimensional Inference Method for Efficient Design. One notable feature of this neural network is the use of a Global Average Pooling layer instead of a Fully Connected (Dense) layer to preserve spatial information (Lin *et al.*, 2013). The model also uses Spatial Dropout rather than standard dropout to help enforce this relationship. The model also incorporates Spatial Dropout for enforcing spatial relationships. See Supplementary Fig. 1 for a diagrammatic overview of the architecture.
- **TIMED_Unbalanced (CNN)**: this model is similar to TIMED but is trained without the balancing operation at each epoch.
- **TIMED_Polar and TIMED_Charge (CNN)**: these models are built on the TIMED architecture and include an additional channel in the frame to specify the polarity or charge, respectively, in addition to the atomic channels. The polarity is based on a Zimmerman score, of less than 20 for non-polar, (-1) and polar (+1) otherwise. The charge is encoded as −1, 0 or +1 depending on the charge of the amino acid (Zimmerman *et al.*, 1968). Both features are encoded at the location of the C$\alpha $ atom of the backbone in an separate channel of the input data.

We used Keras to build the CNN models for ProDCoNN, DenseCPD, DenseNet, TIMED (Chollet, 2015). We trained the models for 50 epochs. Training was performed using a combination of the Cambridge Service for Data-Driven Discovery (CSD3) (NVIDIA Tesla P100 16GB GPU and 36 cores) and our internal servers (Intel Core i9-10980XE CPU @ 3.00GHz with 36 cores and NVIDIA Quadro RTX 8000 48GB GPU). We used Weights & Biases for experiment tracking and hyper-parameter sweeps (Biewald, 2020).

### Validating using PDBench and AlphaFold

To compare our novel models with those of the literature, we used the PDBench toolkit with its benchmark set (Castorina *et al.*, 2023) and AlphaFold 2 (AF2). (Jumper *et al.*, 2021). The metrics were then broken down by fold type. The benchmark set consists of a fold-balanced set of 595 protein structures classified into three main fold types: Mainly $\alpha $, Mainly $\beta $, $\alpha $-$\beta $ folds. Performance metrics for each model were evaluated overall and separately for each fold type. We used AF2 to fold the sequences predicted by the models. To alleviate the computational demands of AlphaFold, we evaluated shape metrics on 10% of the PDBench structures. We used PyMOL structural alignment command ‘cealign’ to calculate RMSD by comparing the original (crystal) structure and the AF2-predicted structure (Schrödinger, 2015).

#### Sequence Metrics

PDBench calculates sequence metrics such as Macro-Recall, and Mean Absolute Error (MAE) for charge and isoelectric point. Macro-Recall is an accuracy metric that accounts for the class imbalance of amino acids: 


\begin{align*} & \text{Macro-Recall} = \sum_{\text{classes}} \frac{\text{ recall of class}}{\text{ number of classes }} \end{align*}

The amino acid composition in proteins varies significantly across different protein folds. Macro-Recall ensures that the maximum accuracy for each amino acid is capped at 1/20 (5%).

MAE measures the average difference in charge and isoelectric point between the original and predicted sequences: 


\begin{align*} & \text{ MAE }=\frac{\sum_{i=1}^{N}\left|y_{i}-\hat{y}_{i}\right|}{N} \end{align*}

where $y_{i}$ represents the charge or isoelectric point for the original sequence, and $\hat{y}_{i}$ represents the predicted value. $N$ is the number of sequences analyzed.

#### Fold recovery

We used AF2 (Jumper *et al.*, 2021) to predict the 3D structure of the predicted sequence. We used root mean squared deviation (RMSD) to compare the distance between the C$\alpha $ of the original crystal and the predicted structure: 


\begin{align*} & \operatorname{RMSD}=\sqrt{\frac{\sum_{i=1}^{n}\left(y_{i}-\hat{y}_{i}\right)^{2}}{n}} \end{align*}

We normalise RMSD for the length of the protein ($n$) using $\operatorname{RMSD}_{100}$ proposed by Carugo & Pongor (2008): 


\begin{align*} & \operatorname{RMSD}_{100}=\frac{\operatorname{RMSD}}{1+\ln \sqrt{\frac{n}{100}}} \end{align*}

To reduce the computational burden of AF2, we ran it on a subset of 59 randomly selected monomeric protein structures from the PDBench set (approximately 10% of the benchmark) covering Mainly $\alpha $, Mainly $\beta $ and $\alpha $-$\beta $ folds.

We predicted the residue sequences using each of the models described in Section 2.3. Subsequently, we folded the predicted sequences using AF2 to obtain the predicted 3D structure. We then calculated the RMSD between the original and the predicted structure for each model and for each fold type. We excluded structures with of the ‘special fold’ as they are highly irregular.

We used a local version of ColabFold (Mirdita *et al.*, 2021) called LocalFold. We used the CEalign command in PyMOL to calculate RMSD because of its robustness to low sequence similarity (Schrödinger, 2015).

### Monte-Carlo-based sampling of amino acid probability distributions

To generate final sequences we took the most likely amino acid at each position (argmax), but we also explored sampling from the sequence probability distributions using a Monte-Carlo-based method. In this case, the temperature affects the chance of selecting low-probability amino acids. As the temperature increases, the sequences become more random. The temperature was applied to the output probability distributions using the following equation: 


\begin{align*} &\frac{\exp \left(z_{i} / T\right)}{\sum_{j} \exp \left(z_{j} / T\right)}\end{align*}

where $T$ represents the temperature, $j$ is the number of classes and $z_{i}$ denotes the predicted probability distribution for class $i$. A temperature of $T=0$ corresponds to selecting the class with the highest probability (argmax), $T=1$ maintains the original distributions and $T>1$ leads to a more uniform (higher entropy) prediction across all classes.

We sampled 20 sequences from the probability distribution of each model for 57 randomly sampled proteins at temperatures 0.2, 0.6 and 1. We then used all five models of AF2 to fold the predicted sequences. Subsequently, each predicted structure was relaxed using the AMBER force field (Salomon-Ferrer *et al.*, 2013). The process resulted in approximately 8K relaxed PDB files, excluding any failed structures due to out of compute errors. The calculations were performed using the CSD3 with NVIDIA A100 GPUs.

We analysed the following metrics: packing density of the original and predicted structures, RMSD, AlphaFold lDDT, Shannon entropy and accuracy. The packing density for the predicted structure was calculated using ISAMBARD as the number of non-hydrogen backbone atoms within a radius of 7 Å (Wood *et al.*, 2017). RMSD was calculated using PyMOL and the alignment function ‘cealign’ (Schrödinger, 2015). Shannon entropy was calculated using SciPy and NumPy based on the probability distribution output of the models (Virtanen *et al.*, 2020). The maximum entropy can be computed using the formula: $\log _{2} N_{\text{classes}}$. For models predicting 20 amino acid classes, the maximum entropy is 4.32.

The full list of structures analysed is as follows: *1k5cA, 1kapP, 1dmlA, 1bx7A, 1igqA, 1jh6A, 1k5nA, 1c3mA, 1gp0A, 1jkeA, 1muwA, 1c1yB, 1b2pA, 1i7wB, 1a92A, 1a41A, 1devB, 1cruA, 1l0sA, 1iz5A, 1jofA, 1gprA, 1luzA, 1lpbA, 1ewfA, 1b8kA, 1hf2A, 1jm1A, 1kcfA, 1j5uA, 1jdwA, 1gxmA, 1lktA, 1lslA, 1io0A, 1h70A, 1itvA, 1k4zA, 1dvoA, 1hxrA, 1hq0A, 1j3aA, 1b77A, 1g3pA, 1kkoA, 1chdA, 1i4uA, 1genA, 1i4jA, 1ejdA, 1gppA, 1dqgA, 1flgA, 1jovA, 1g61A, 1h32A, 1ds1A*.

### TIMED-Design: tooling and user interface

TIMED-Design is an open-source Python repository that provides a user interface (UI) and various tool kits for the usage, analysis, and visualization of protein sequence design models. It can be used with any CNN model that takes frames as input, such as TIMED, DenseNet, DenseCPD, ProDCoNN.

TIMED-Design includes a responsive UI created with Streamlit. The UI allows the selection of a PDB file from the Protein Data Bank or an option for uploading a file. Proteins are voxelised into frames using Aposteriori and predicted with the chosen model. The UI offers the following features:

- Metrics such as charge, isoelectric point, molecular weight and composition.
- Visualization of prediction probabilities distributions on the 3D structure of the protein.
- Performance plots, including precision/recall, prediction bias and sequence logo.
- Monte Carlo sampling at different temperature factors to generate novel sequences based on the probability distribution output of the CNN models.
- Confusion matrix between the original and the predicted amino acids.
- Prediction bias plot for the original and predicted sequence against the natural frequency of amino acids.

## Results

### CNN models and the state of the art

**Fig. 2:**
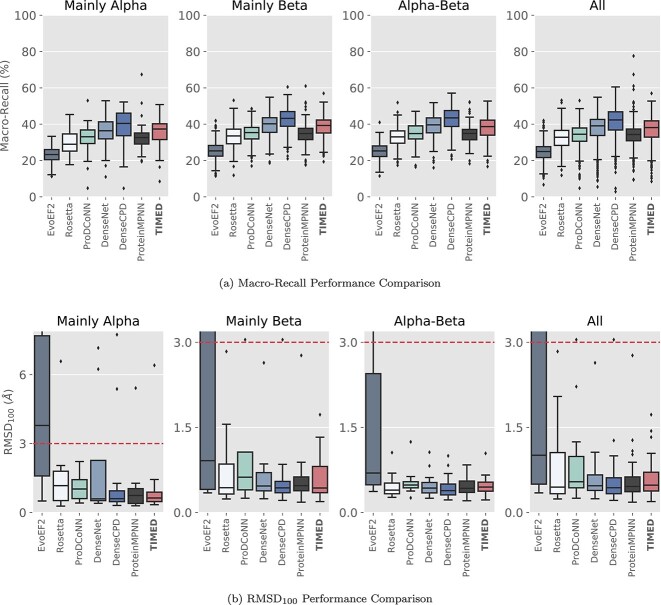
Performance metrics of physics and DL models. (**a**) Macro-Recall, sequence accuracy resistant to class imbalance of amino acids. (**b**) Fold recovery as determined by RMSD of the template structure to the AlphaFold model, normalized by the protein length. All plots are separated by fold type.

To assess the performance of the TIMED family of models, we compared them to other DL-based sequence design algorithms as well as some physics-based methods. For the CNN-based models, we took the most likely residue at each position to generate the final sequence. We compared performance using a range of accuracy metrics, as well evaluating recovery of the template fold by measuring the RSMD between an AlphaFold 2 model of the designed sequence with the template structure. On average, DL models outperform the physics-based methods (EvoEF2 and Rosetta) on accuracy and fold recovery metrics (Fig. 2). Overall, the performance of the CNN-based methods was higher than that of the GNN-based ProteinMPNN, although it is important to note that the reported performance is lower than the value reported in the original ProteinMPNN paper. In order to directly compare the architectures, all models in this paper were trained with the same training set, including ProteinMPNN, which led to a drop in performance. The DenseCPD architecture, which we reimplemented and made available, has the highest overall macro-recall, although fold recovery was similar across all the CNN- and GNN-based models.

### Balancing amino acids and prediction bias

We observed that balancing amino acids at training time (TIMED vs TIMED_Unbalanced) increases accuracy slightly though it has little effect on macro-recall. However, we wanted to determine whether balancing leads to biases in the selection of amino acids in the predicted sequences (Supplementary Fig. 11).

When TIMED and ProDCoNN were trained without balancing, while there was no change in macro-recall, the raw accuracy of sequence recovery on the benchmarking set increased by 6.32 and 6.90%, respectively. However, this also led to an increased prediction bias for the most common amino acids. The increased prediction bias is particularly prominent for alanine, glutamate and leucine in $\alpha $-helices (4, 10 and 6% bias in TIMED; 2, 15 and 10% in ProDCoNN, respectively) and, to a lesser extent, leucine and valine in $\beta $-sheets (3 and 5% in TIMED, and 4 and 7% in ProDCoNN).

When the amino acids were balanced through random undersampling, the prediction bias for all amino acids approached 0%, indicating that the predicted and true sequences have similar amino acid distributions.

### Incorporating polarity and charge as design constraints

In typical protein design settings, designers often have specific constraints and requirements for the proteins they are designing, such as the incorporation of cofactor binding sites, preservation of active site residues or retention of charge compatibility. One powerful aspect of the voxel-based representation of proteins used by the CNN models presented here is that they can incorporate these constraints into the protein design process simply by adding channels that encode additional information to the input frame.

Here, we investigate the effect of incorporating polarity and charge as separate channels in the input frame. We compare the performance of TIMED with TIMED_Polar and TIMED_Charge. All models share the same underlying architecture. However, The TIMED_Polar and TIMED_Charge models receive additional polarity or charge information as input.

**Fig. 3:**
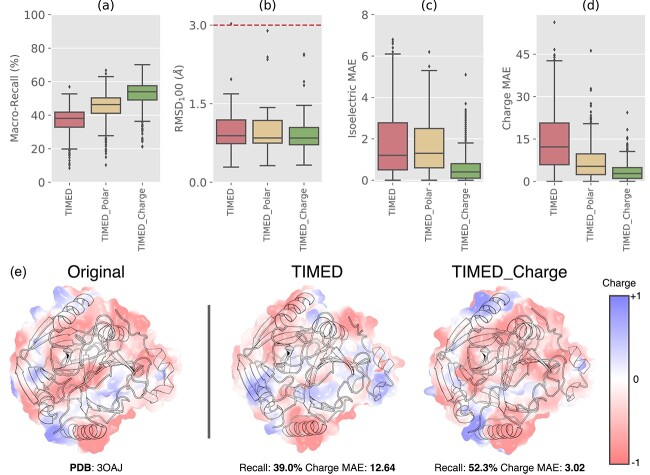
Performance metrics of TIMED, our CNN model (red) and its variants TIMED_Polar (yellow) and TIMED_Charge (green). (**a**) Macro-Recall performance, sequence accuracy resistant to class imbalance of amino acids. (**b**) AlphaFold RMSD normalized by the protein length. (**c**) and (**d**) show the MAE for Isoelectric Point and Charge, respectively. See Supplementary Section 2 for these plots by fold and with other models. (**e**) Charge and Recall performance comparison of TIMED and TIMED_Charge model for PDB: 3OAJ. Both models correctly recover the target structure to under 1 Å RMSD; however, TIMED_Charge maintains charges and achieves higher sequence recall.

As Fig. 3 and Supplementary Figs 6–9 show, the TIMED_Polar and TIMED_Charge models outperform the TIMED model in terms of Macro-Recall. Notably, the TIMED_Charge model achieves significantly better performance across all metrics. The TIMED_Polar generally outperforms the TIMED model in all metrics, except in the AlphaFold RMSD of Mainly $\alpha $-helical folds where TIMED has a smaller range of RMSD values (Supplementary Fig. 7).

Comparing TIMED with TIMED_Charge, we observed that, while most designs exhibit similar RMSD values, the charge model better maintains overall charges, evident by the significantly lower charge MAE and higher recall achieved by the TIMED_Charge model. Figure 3(e) shows a protein with an equal RMSD of 1.05 Å for both models, while TIMED_Charge is better able than TIMED at effectively preserving areas of charges.

### Performance and dependence on resolution

**Fig. 4:**
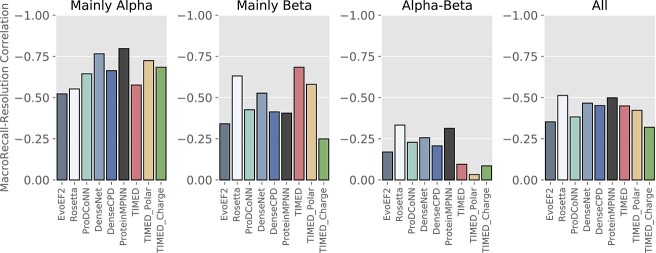
Pearson correlation coefficients between Macro-Recall and resolution for models over different types of folds.

We investigated the correlation between Macro-Recall Performance and Resolution per fold in Fig. 4. Among the DL models, the Mainly $\alpha $ folds exhibited the highest correlation between Macro-Recall and Resolution. In contrast, the physics models demonstrated a slightly stronger correlation between resolution and Macro-Recall in the $\beta $ folds compared to the $\alpha $-helical fold. The $\alpha $-$\beta $ folds generally exhibited low correlations.

Finally, we compared the overall correlation of performance between models, as shown in the Supplementary Fig. 12. The DL models were highly correlated among themselves. ProteinMPNN had the highest correlation with TIMED, while all CNN models had similar correlations between each other, indicating that the basis of predictions might be different between these models.

### Monte Carlo sampling for sequence generation

Once a probability distribution for amino acid identity has been generated for all amino acids in a protein, a sequence can be generated by drawing from these distributions. A naive approach would be to take the most probable amino acid at each position, but we could generate many more sequences by sampling from these distributions. We used TIMED-Design to perform weighted random sampling from the predicted probability distributions, with a temperature factor to increase sequence variability. Further to this, we hypothesised that the higher the performance of the model, the more robust sequence generation should be to higher temperatures.

To introduce diversity in the sampled sequences, we applied different temperature factors (0.2, 0.6 and (1) when sampling from the probability distributions then used AF2 to predict the structures of the sampled sequences. The following metrics were analyzed: Accuracy, Entropy, Mean Packing Density of the predicted structure, AlphaFold lDDT and RMSD using PyMol (Schrödinger, 2015). Entropy was calculated using the Shannon Entropy function of SciPy (Virtanen *et al.*, 2020). In Supplementary Table 1 and 2, we report the number of structures and average metrics.

#### Correlation trends between sequence and fold recovery at different temperatures

**Fig. 5:**
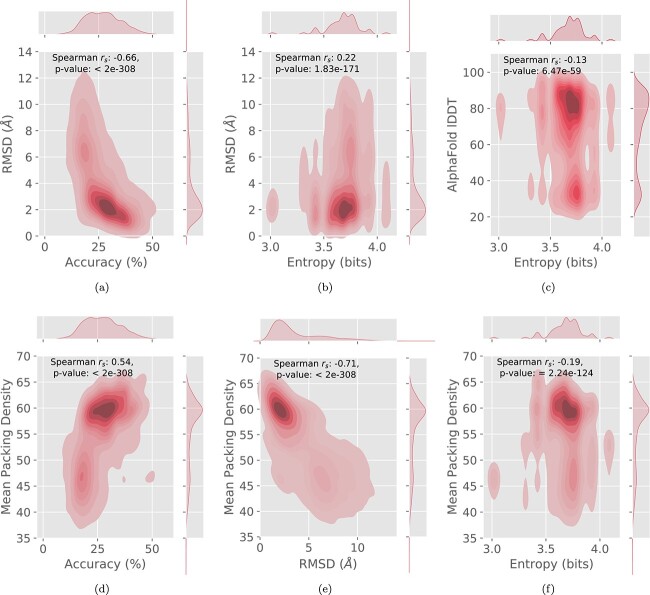
RMSD performance against Accuracy (**a**) and Entropy (**b**), AlphaFold local distance difference test (lDDT) against Entropy (**c**), and Mean Packing Density of predicted structure against Accuracy (**d**), RMSD (**e**) and Entropy (**f**) for TIMED averaged across sampling temperatures (0.2, 0.6, 1). Sequences were sampled from the probability distribution of TIMED at different temperatures. The 3D shape of the predicted sequences was then computed through AlphaFold 2 and RMSD was calculated between the predicted shape and the original shape of each protein.

First, in Fig. 5(a), we investigated the correlation between the accuracy of the TIMED model and the RMSD of the sampled structure by sampling sequences at different temperatures. We reasoned that, as we increase the temperature, the RMSD between the predicted structure of the designed sequence and the template structure would increase, but this behaviour might not be uniform across models. We calculated the Spearman correlation coefficient for accuracy and RMSD values and report the Spearman coefficients and corresponding $P$ values for each fold and temperature in Supplementary Table 2. We observed that higher accuracy is generally associated with lower RMSD, although the strength and significance of the correlation varies depending on the fold and temperature. All correlations had very significant $P$ values.

Next, we examined the correlation between the average prediction Shannon entropy of the output sequence probability distribution and RMSD, as illustrated in Fig. 5(b). Generally, higher entropy values were weakly correlated with higher RMSD, which makes sense as the Shannon entropy is roughly equivalent to the confidence of the amino acid identity at each position. The correlation is significant across all folds and is strongest in the mainly Mainly $\alpha $ fold and $\alpha $-$\beta $ and weaker in the Mainly $\beta $.

Finally, in Fig. 5(c), we investigated the correlation between the average prediction entropy and the AlphaFold lDDT. There was no clear correlation between these values.

#### Correlation trends between performance and packing density

We also investigated the relationship between the Packing Density of the predicted structure and RMSD, Accuracy, and Prediction entropy at temperatures of 0.2, 0.6 and 1. We avoided temperatures higher than 1 as increasing the temperature further would results in random-like sequences and predicted structures with very high RMSD.

As shown in Fig. 5(d, e, f), there is generally a positive correlation between packing density and performance, indicating that regions of the proteins with higher packing density tend to be predicted with higher accuracy, lower RMSD, and lower entropy. The $P$ value for all of the correlations is very significant for all the performance metrics.

The correlation between Mean Packing Density and Accuracy (Fig. 5d) is significant and positive with a Spearman $r_{s}$ of 0.54. The correlation between Mean Packing Density and RMSD (Fig. 5e) is negative and significant with with a Spearman $r_{s}$ of −0.71. Finally, there is a weak correlation between Mean Packing Density and entropy (Fig. 5f) is negative and significant with a Spearman $r_{s}$ of −0.19. Interestingly, as shown in Supplementary Fig. 13, the Mean Packing Density and entropy correlation is much stronger for the Mainly $\alpha $ ($r_{s}$ −0.45) and $\alpha $-$\beta $ ($r_{s}$ −0.43) folds compared to $\beta $ ($r_{s}$ −0.08).

### TIMED-Design: a model-agnostic interface for protein sequence design models

All of the models we have described in this paper are publically available, including re-implementions of the other convolutional networks beyond our own. Furthermore, we created TIMED-Design, an interface for designers to interact with these different sequence design models.

TIMED-Design is an open-source UI and CLI package built with Streamlit and Stmol (Nápoles-Duarte *et al.*, 2022). It currently features ProDCoNN, DenseCPD, DenseNet, TIMED, TIMED_Polar and TIMED_Charge.

#### User interface

**Fig. 6:**
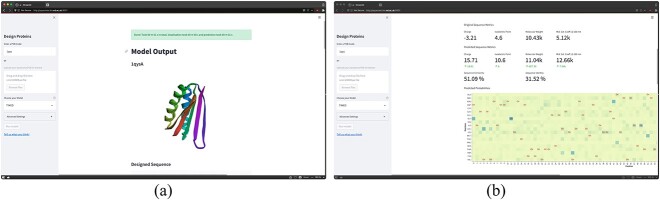
Overview of the TIMED-Design user interface. (**a**) Once a backbone is selected, it is voxelised and predicted by the chosen model. (**b**) Prediction probabilities and designed sequence metrics.

The UI allows user to select a backbone from a PDB code or upload a PDB file. The backbone is voxelised into frames by Aposteriori and the selected model is used to predict the most likely sequence (see Fig. 6a). We display the the most likely residues at each position with a sequence logo. Alternatively, an interactive plot of the predicted probabilities is also available, featuring the original (‘ori’) amino acid coloured in red (Fig. 6b).

The interface features several sequence metrics such as charge and isoelectric point. The probability distributions are displayed as a heatmap and as a sequence logo. Each position can be explored and visualised directly on the 3D shape of the protein.

Other plots include metrics plot (precision, recall and F1 score) per residue, prediction bias and a confusion matrix between the true residue in the protein chain and the predicted residue by the models. Other features include Monte Carlo temperature sampling where the user can specify the number of sequences to sample from the probability distribution.

In the case of TIMED_Polar and TIMED_Charge, the UI allows users to change or fix specific sites in the protein as polar/non-polar or positive/neutral/negative charge. This is helpful in the case of re-design of specific interfaces of the protein. The UI is available at the following URL: https://pragmaticproteindesign.bio.ed.ac.uk/timed

#### Command line interface

The command line interface (CLI) features the same functionalities as the UI as well as additional analysis features involving further analysis and plots. All the scripts used to analyse or generate plots are present in a separate folder to allow customisation.

## Discussion

CNNs offer a flexible and performant architecture to encode spacial information for proteins. In this study, we introduce the TIMED models, which are state-of-the-art DL models for protein sequence design. We demonstrate that the expansion of the input voxel representation to include design-centric information such as polarity and charge, can drastically improve performance. Furthermore, while developing and benchmarking these methods, we made many observations about their performance that is important to consider when they are applied.

### Encoding additional information in the voxel input allows for broader applications for protein re-design

The polar and charge models implemented with the default TIMED architecture demonstrate superior performance and exhibit notable differences in property prediction compared to other models. While these models may not be suitable for truly *de novo* applications, they can be effectively used for protein redesign when both backbone and property information are available as input. Moreover, the UI allows users to selectively modify the property at specific positions, enabling targeted ‘re-painting’ of specific regions of a protein with different properties, such as switching a binding region from positive to negative charge. The improved performance of these models suggests that further work on feature engineering for convolutional models is warranted, where more comprehensive information about the desired function of the protein could be incorporated within the voxel space, such as fixed amino acids in a catalytic site.

##  

### Per-fold metrics for a granular performance overview

There is a notable difference in the performance of all the design models across the different fold classes for both sequence and fold recovery metrics (Fig. 2 and Supplementary Figs S2–S5). Interestingly, all the DL methods generally perform comparably well or better than physics methods when it comes to designing mainly $\beta $ structures, despite $\beta $ structures being historically challenging targets (Huang *et al.*, 2016; Woolfson *et al.*, 2015). Even more surprisingly, Mainly $\beta $ structures show a lower correlation between Macro-Recall and template structure resolution (Fig. 4) than Mainly $\alpha $ structures, so this cannot be explained by Mainly $\beta $ structures requiring higher quality templates.

Additionally, in Fig. 4, we see a strong correlation between Macro-Recall and Resolution, specifically for Mainly $\alpha $ and $\beta $ folds, and to a lesser extent for $\alpha $-$\beta $. For the Mainly $\alpha $, ProteinMPNN exhibits the strongest correlation, followed by DenseNet and TIMED_Polar.

##  

### Balancing amino acids at training time reduces prediction bias

We explored the effect of balancing amino acid classes during training time. In some ways, unbalanced classes better reflect the biochemical availability of the individual amino acids, which could improve production of the proteins in living systems. However, this would mean that the biases of natural proteins would be reflected in the sequences produced by the design algorithms, when there is strong evidence that functional proteins exist in sequence spaces that are unexplored in nature (Weidmann *et al.*, 2019).

##  

### AlphaFold as a tool for sequence design validation

We have discovered that accuracy, macro-recall and other statistical metrics can accurately estimate a model’s performance only up to a certain point. For example, the EvoEF2 and ProDCoNN have similar performance as measured by sequence metrics. However, differences in RMSD in the AlphaFold2 predictions are at times significant, for example, in the Mainly $\alpha $ folds. Although AlphaFold comes at significant computational cost, alternative lighter-weight structure prediction algorithms, such as OmegaFold (Ruidong *et al.*, 2022) or ESMFold (Lin *et al.*, 2022), could be used in its place.

Additionally, most DL models perform similarly in terms of RMSD, usually under 3 Å and differences between models are usually less than 1 Å, which is the median RMSD from the original AlphaFold2 paper (Jumper *et al.*, 2021). Perhaps, after a certain level of performance, differences in accuracy metrics become less relevant. In the case of *de novo* design, for example, high accuracy might limit the utility of the design method, as the sequences produced will have lower variability. In real-world applications of protein, the increased diversity of lower accuracy models might be more desirable, especially when the experimental strategy involves high-throughput screening.

When comparing the performance of ProteinMPNN when trained with the culled PDB set (40K chains) and its performance using the full PDB (500K+ chains) we see a 33% increase in performance Dauparas *et al.* (2022a). It is evident therefore that the performance of most models is sensitive to the amount of training data. Selecting larger portions of training data would lead to higher sequence recovery. However, as all of these models achieve RMSD scores well below 3 Å, it is possible that training with smaller portions of the data set could be a way to obtain more ‘creative’ sequence designs, i.e. sequences with similar shapes but significantly different sequence similarity, although this requires further investigation.

##  

### Monte Carlo sampling to produce sequences

The output of DL models for protein sequence design is a probability distribution for each amino acid of the template structure. In the case of the CNNs, the prediction of each position is independent of the next. This means that selecting the highest probable amino acid for each position may not be the best strategy for selecting sequences. An alternative approach is to sample from the probability distribution and generating many sequences.

In Fig. 5(b) and (c), we observed that the Shannon entropy of the output probability distributions correlates with RMSD and AlphaFold lDDT. This observation suggests that Shannon entropy could be used as a confidence score for sequence predictions in this case. Higher entropy values indicate more randomness in the distributions, indicating less confidence in the model’s predictions. Calculating prediction entropy is significantly faster than running AlphaFold on the sequence, allowing high-entropy regions to be identified as candidates for further optimization. Futhermore, the Shannon entropy might be indicative of the quality of the backbone template and used as a basis for improvement of the template without computationally expensive simulation.

##  

### Performance at different packing density varies with fold

We observe that performance, as measured by metrics such as RMSD and accuracy, varies at different packing densities (see Fig. 5 and Supplementary Fig. S13). Generally, higher packing density is correlated with better performance in the Mainly $\alpha $ and $\alpha $-$\beta $ folds. This correlation can be attributed to the fact that high-density regions often correspond to core of the protein, which are typically composed of hydrophobic residues (Banach *et al.*, 2020). Similar observations have been made in ProteinMPNN (Dauparas *et al.*, 2022a). Additionally, the reduced mobility of residues within hydrophobic cores, compared to solvent-exposed residues, contributes to a more well-defined backbone conformation. Interestingly, we find that the strength of these correlations varies across different folds. For instance, mainly $\beta $ folds exhibit a weaker correlations compared to mainly $\alpha $ and $\alpha $-$\beta $ folds in terms of accuracy and entropy. This highlights the influence of fold-specific characteristics on the relationship between packing density and performance metrics.

##  

### TIMED-Design: UI and CLI

The TIMED-Design UI bridges the gap between designers and methods developers. The goal for the UI is to remove all the complexity involved in installing the models, the environment, and interpreting the predictions. To the best of our knowledge, TIMED-Design is the first model-agnostic UI for non-technical people to interact with state-of-the-art protein sequence design models. The CLI also offers users the ability to create scripts to further interact with these models programmatically. In future, we aim to incorporate TIMED-Design into DE-STRESS (Stam & Wood, 2021), our platform for evaluating protein designs, so that new designs can be generated, evaluated and shortlisted, all in one application.

## Conclusion

In this paper, we have demonstrated that CNNs are a powerful and flexible architecture for protein sequence design. We described the development and benchmarking of a range of high-performance sequence design algorithms, as well as the reimplementation of other CNNs from the literature. We have shown that voxelised representation of protein structure information is versatile and enables the incorporation of additional design considerations such as charge. Finally, we provided a public implementation of a few models and integrate them into a web-based design tool for insightful exploration and comparison.

## Supplementary Material

PEDS_TIMED_Design_supplementary_updated_gzae002
