# Supplementary material for: TIMED-Design: flexible and accessible protein sequence design with convolutional neural networks
Source: Protein Eng Des Sel. 2024 Jan 30;37:gzae002. doi: 10.1093/protein/gzae002 (PMC10939383; doi:10.1093/protein/gzae002)
Supplement: PEDS_TIMED_Design_supplementary_updated_gzae002 [file peds_timed_design_supplementary_updated_gzae002.pdf]

TIMED-Design: Flexible and Accessible Protein Sequence Design with Convolutional Neural Networks

Supplementary Materials

1 TIMED Architecture

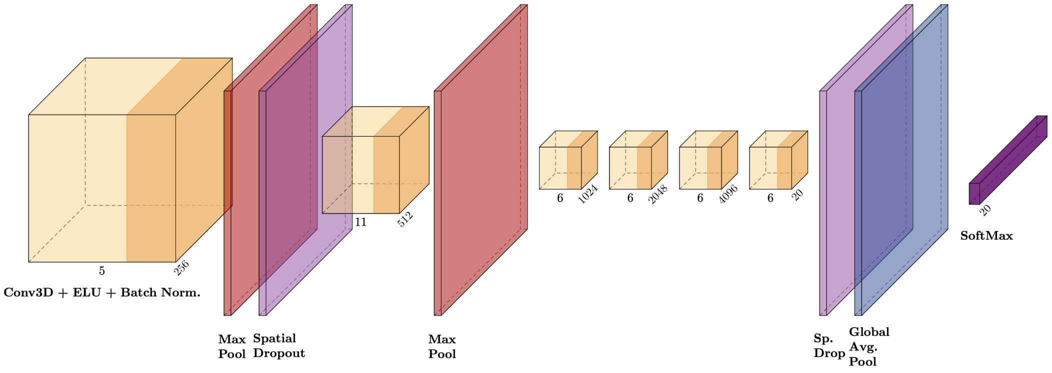

Figure S1: Architecture of the TIMED family of neural networks.

2 Charge and Polar Performance Comparison

2.1 All Models

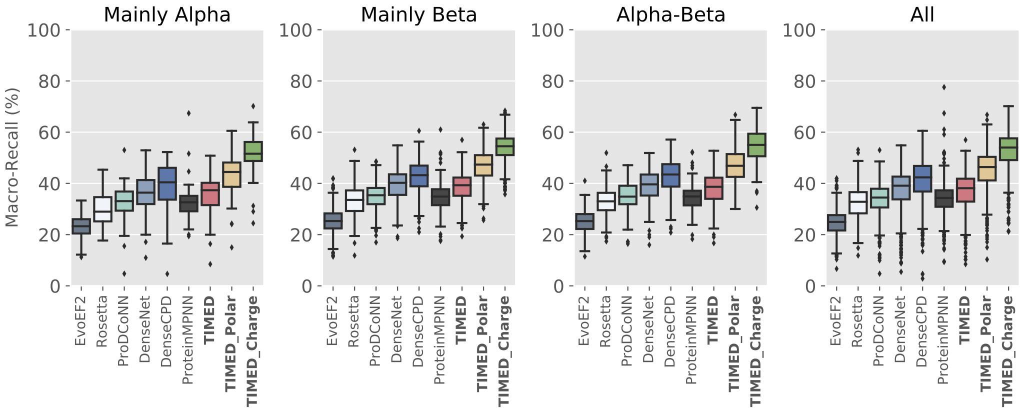

Figure S2: Macro-Recall Performance Comparison

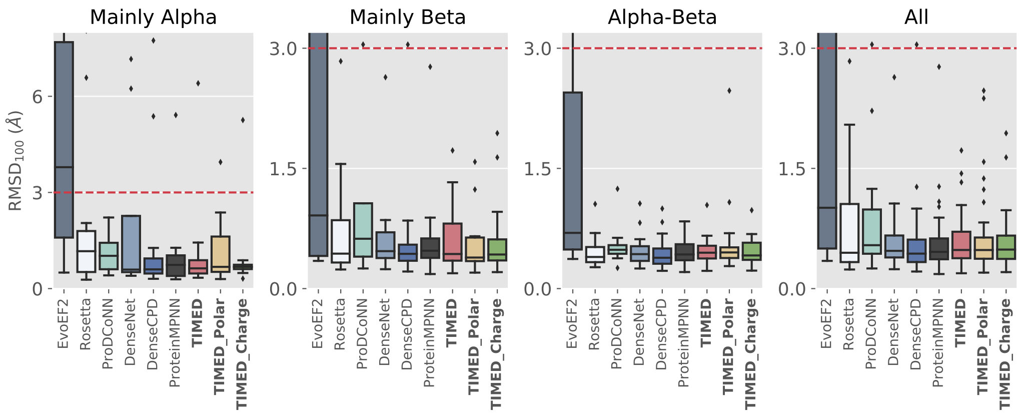

Figure S3: $RMSD_{100}$ Performance Comparison

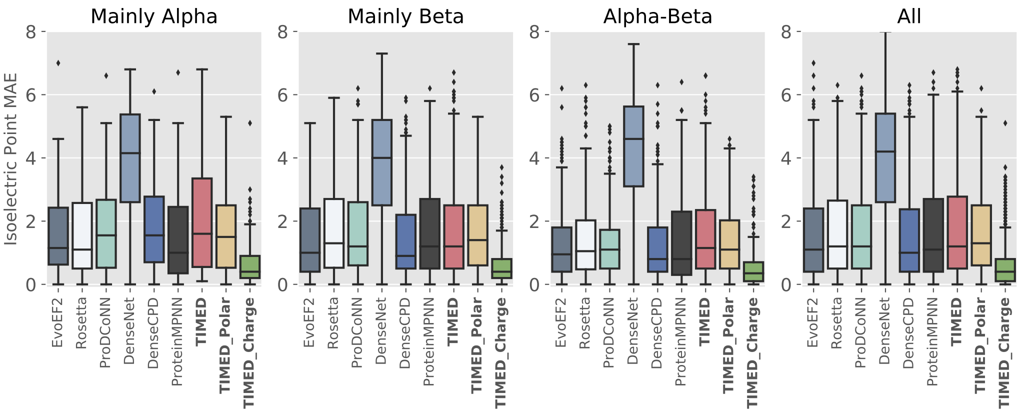

Figure S4: Isoelectric Point Performance Comparison

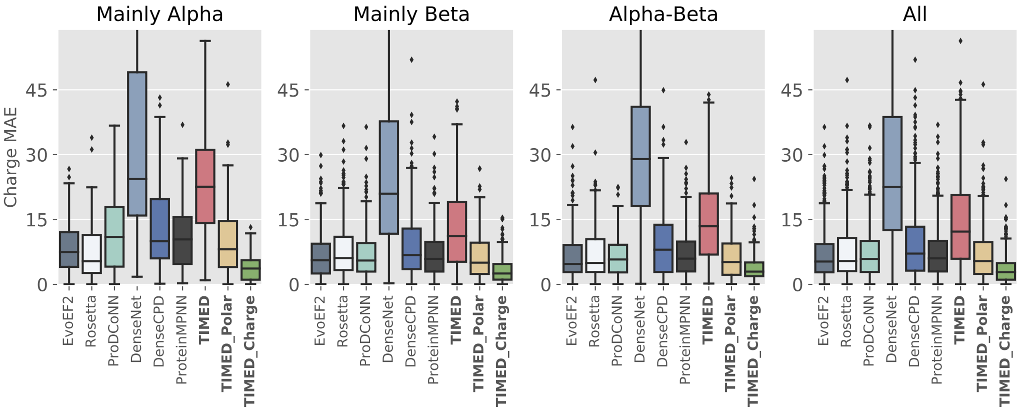

Figure S5: Charge Performance Comparison

2.2 TIMED Models Only

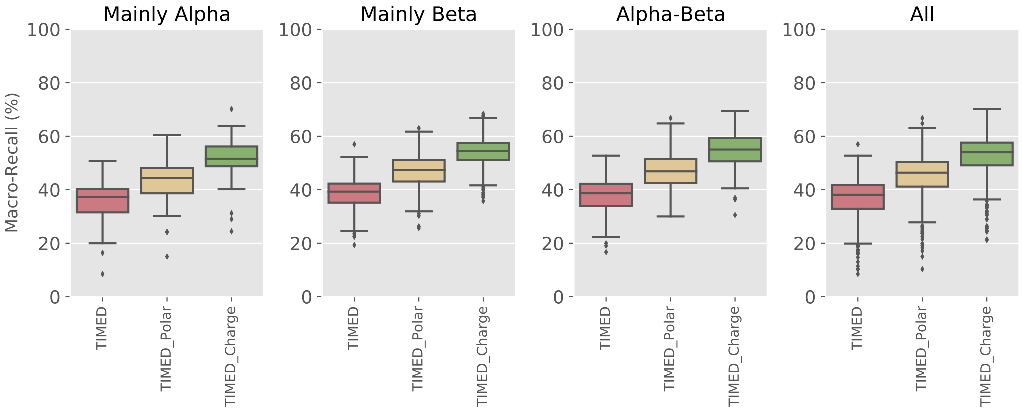

Figure S6: Macro-Recall Performance Comparison

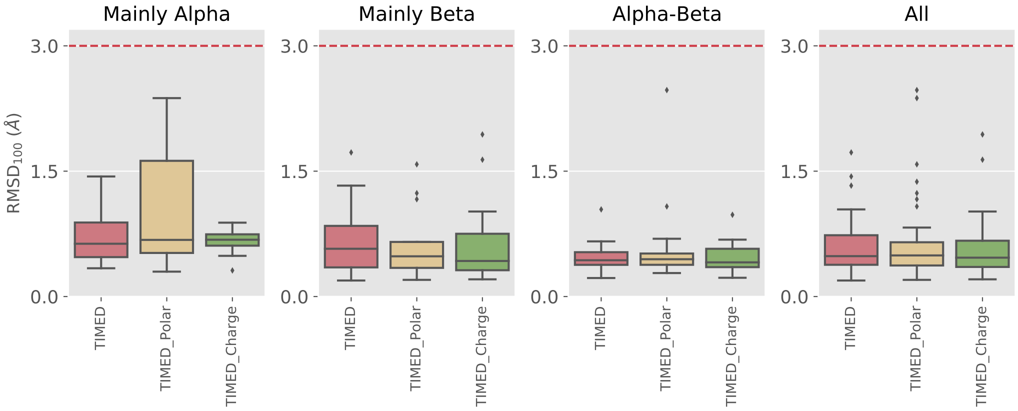

Figure S7: RMSD_{100} Performance Comparison

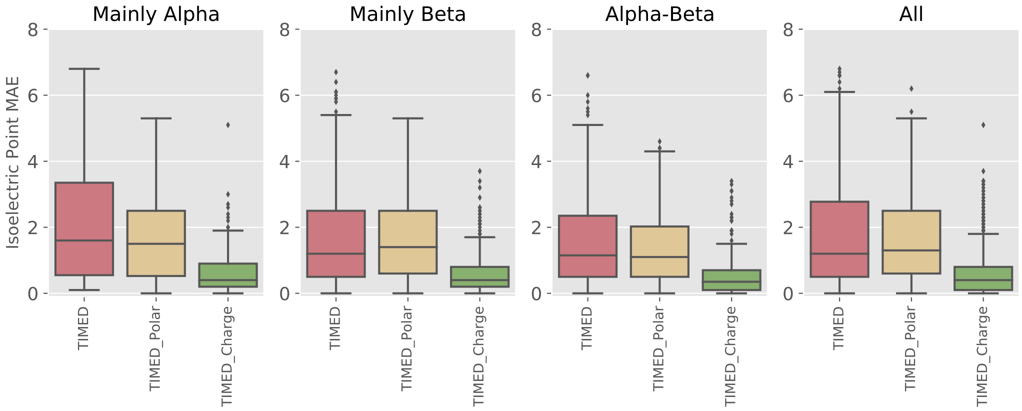

Figure S8: Isoelectric Point Performance Comparison

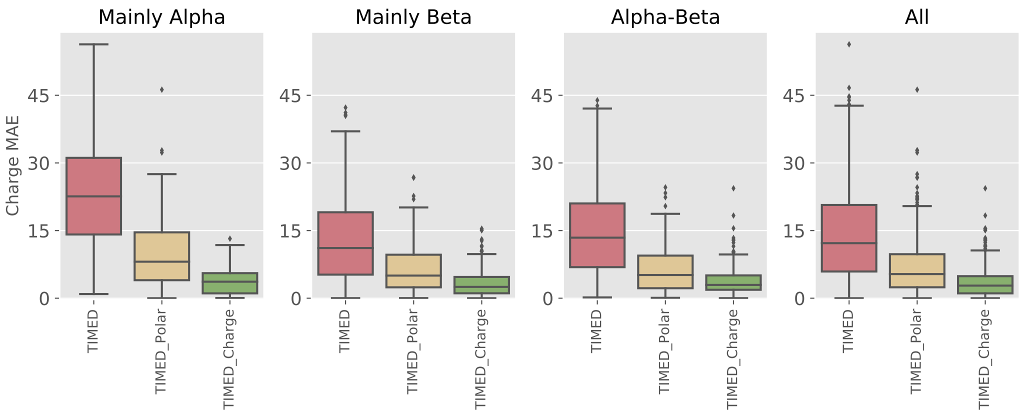

Figure S9: Charge Performance Comparison

3 Retraining Models for Performance Comparison

Models in the literature use different training and validation data, making fair performance comparisons impossible. For this reason we retrained all of the state-of-the-art deep learning models with the same dataset. For benchmarking we used the PDBench structures Castorina et al. [2023] which were removed from the training set.

As several CNN models are not publicly available, we reached out to the authors of ProdCoNN Zhang et al. [2020] and DenseCPD Qi and Zhang [2020] to obtain their neural architecture. We then trained all of the CNN models with the same dataset.

For ProteinMPNN, we had to re-create tooling around custom dataset creation under instructions of the authors, as this code had not been recorded. We first recreated the training set to ensure that the performance was comparable to that reported in the paper. We then retrained ProteinMPNN with the same structures as the CNN models. Tooling, scripts, datasets, training curves, and trained models are available in this repository: `https://github.com/wells-wood-research/ProteinMPNN_custom_training`.

4 Unbalanced Performance Comparison

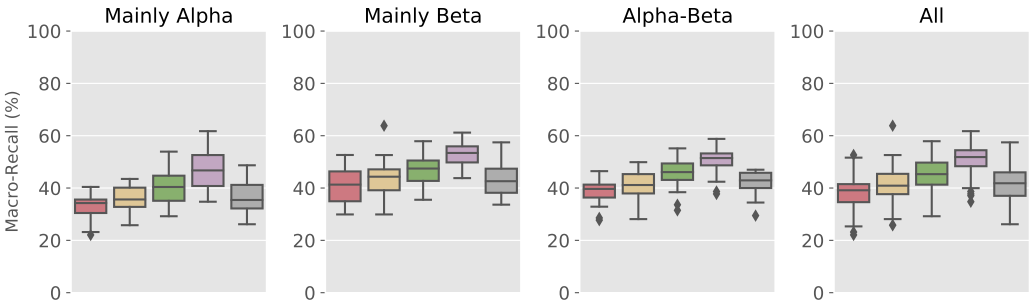

(a) Macro-Recall Performance Comparison

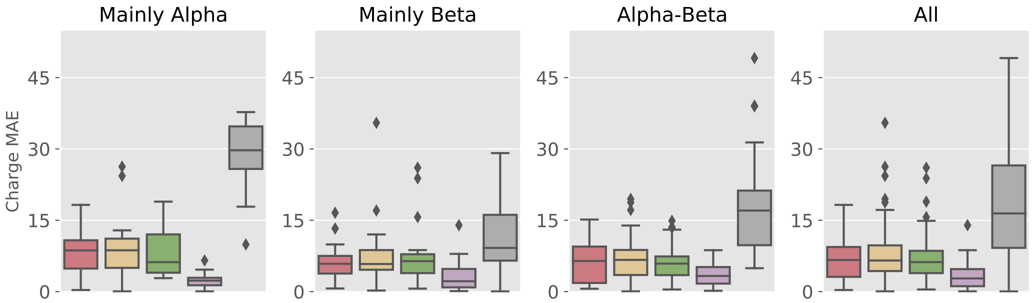

(b) Charge Performance Comparison

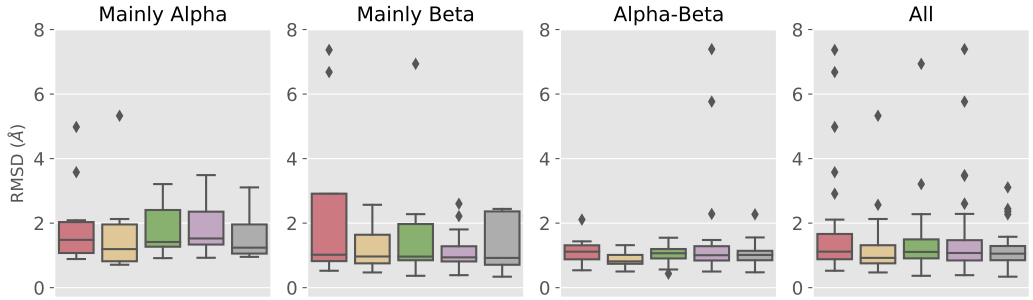

(c) RMSD_{100} Performance Comparison

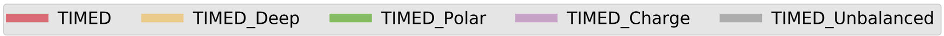

Figure S10: Performance of TIMED networks with unbalanced classes in training data.

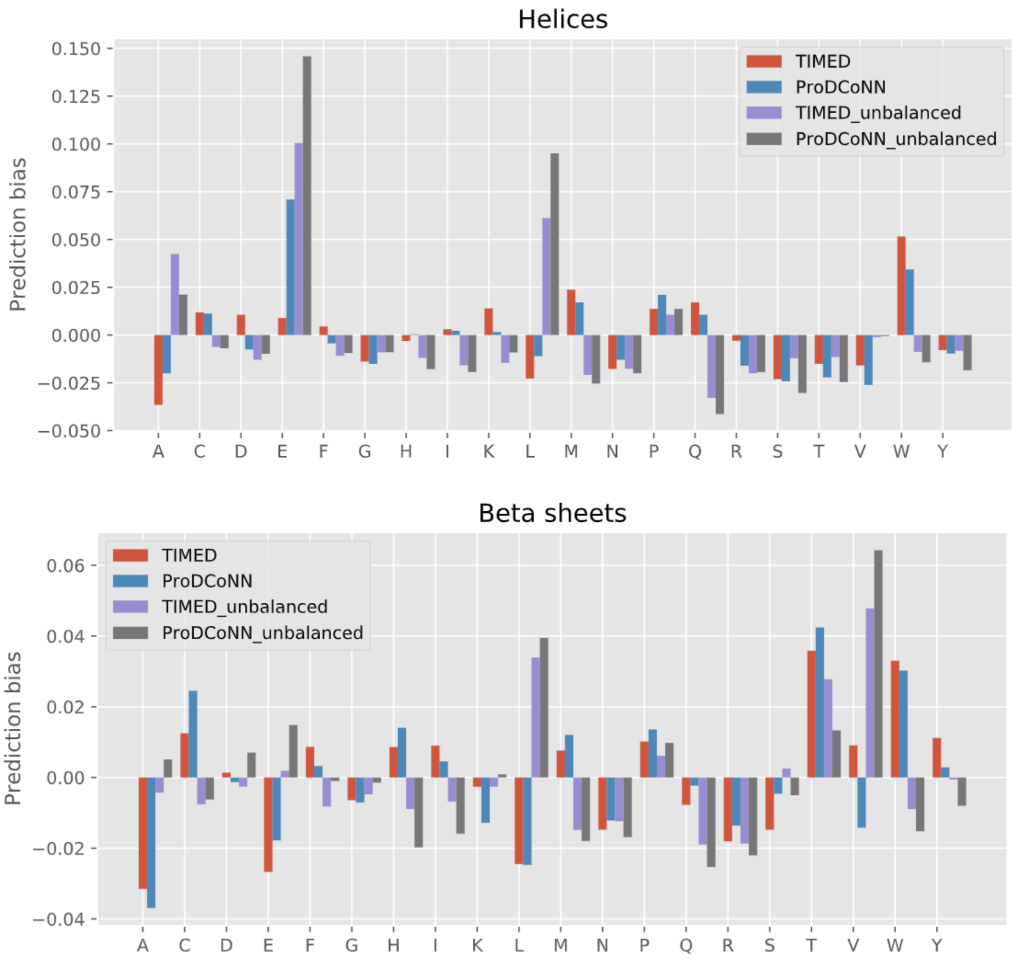

Figure S11: Prediction bias comparison for TIMED and ProDCoNN models both balanced and unbalanced versions.

5 Performance Correlation

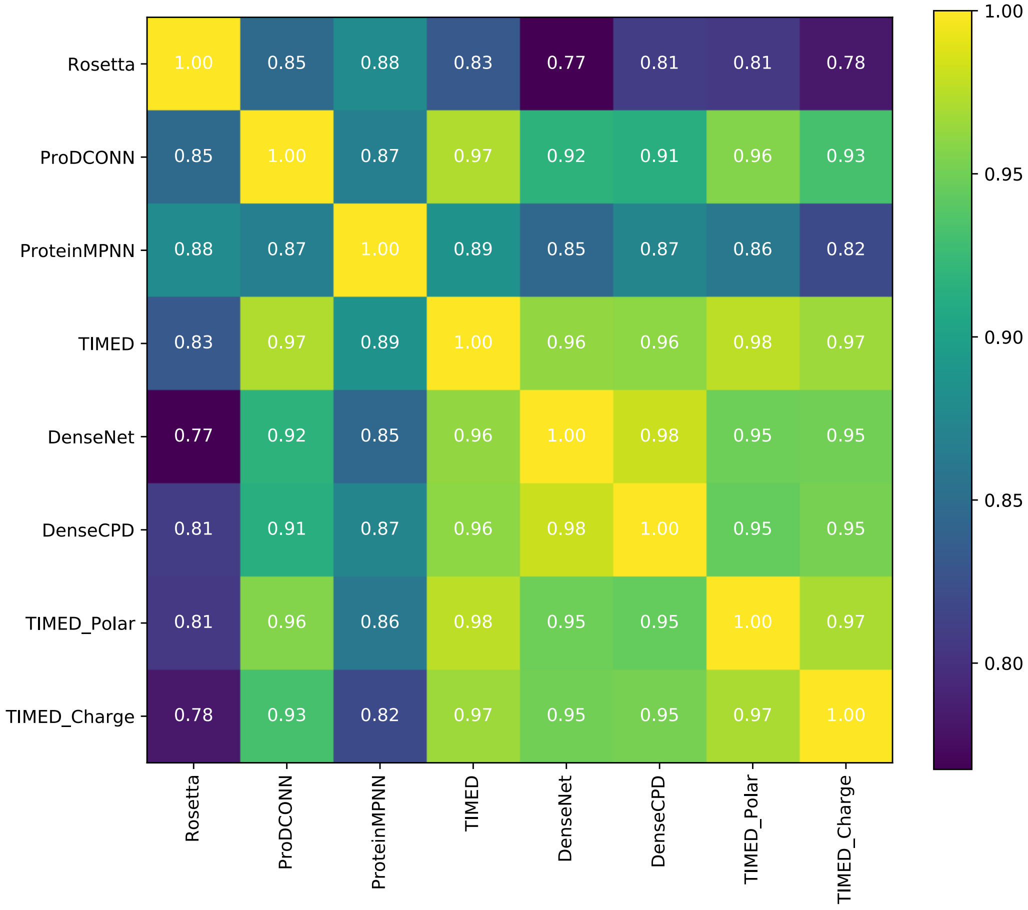

Figure S12: Performance Correlation Matrix between all models tested

6 Monte Carlo Sampling Data

Class	Temperature	n	Accuracy	Entropy	Packing Density	AlphaFold lDDT	RMSD
Mainly Alpha	0.2	3800	33.15	3.75	66.18	87.99	1.91
Mainly Alpha	0.6	3800	25.67	3.75	66.10	70.54	3.80
Mainly Alpha	1.0	3800	18.46	3.75	66.10	43.30	6.44
Mainly Beta	0.2	25830	35.62	3.61	63.56	82.17	2.44
Mainly Beta	0.6	27550	28.33	3.61	63.60	69.06	3.54
Mainly Beta	1.0	27055	20.92	3.61	63.59	45.30	5.94
Alpha-Beta	0.2	19000	34.23	3.73	64.36	87.33	1.92
Alpha-Beta	0.6	19950	26.82	3.73	64.51	75.21	2.83
Alpha-Beta	1.0	19000	19.19	3.72	64.50	46.27	5.79

Table 1: Monte Carlo sampling information.

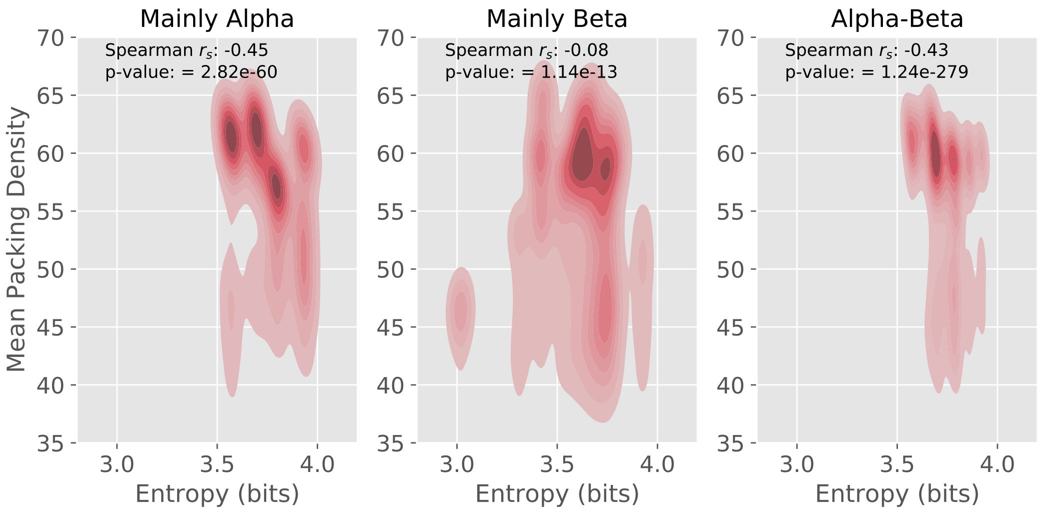

Figure S13: Mean Packing Density and Entropy over Different Folds

References

Leonardo V Castorina, Rokas Petrenas, Kartic Subr, and Christopher W Wood. PDBench: evaluating computational methods for protein-sequence design. *Bioinformatics*, 39(1):btad027, 01 2023. ISSN 1367-4811. doi: 10.1093/bioinformatics/btad027. URL `https://doi.org/10.1093/bioinformatics/btad027`.

Yifei Qi and John Z. H. Zhang. DenseCPD: Improving the accuracy of neural-network-based computational protein sequence design with DenseNet. *Journal of Chemical Information and Modeling*, 60(3):1245–1252, mar 2020.

Yuan Zhang, Yang Chen, Chenran Wang, Chun-Chao Lo, Xiuwen Liu, Wei Wu, and Jinfeng Zhang. ProDCoNN: Protein design using a convolutional neural network. *Proteins: Structure, Function, and Bioinformatics*, 88(7):819–829, jan 2020.

Fold	Temp.	Var 1	Var 2	Pearson Coeff	p value	Spearman Coeff	p value
All	All	Accuracy	RMSD	-0.56797	0	-0.655642	0
Mainly Alpha	0.2	Accuracy	RMSD	-0.595841	2.52517e-39	-0.350521	7.32416e-13
Mainly Beta	0.2	Accuracy	RMSD	-0.124207	6.84963e-11	-0.233826	2.41201e-35
Alpha-Beta	0.2	Accuracy	RMSD	-0.481453	2.79076e-116	-0.603338	4.02228e-198
Mainly Alpha	1.0	Accuracy	RMSD	-0.37723	5.65311e-15	-0.446121	5.86543e-21
Mainly Beta	1.0	Accuracy	RMSD	-0.407154	7.5916e-115	-0.446668	1.49708e-140
Alpha-Beta	1.0	Accuracy	RMSD	-0.479247	1.18128e-115	-0.510681	1.28883e-133
Mainly Alpha	0.6	Accuracy	RMSD	-0.672088	6.87584e-54	-0.562392	9.7606e-35
Mainly Beta	0.6	Accuracy	RMSD	-0.32244	3.78564e-71	-0.333515	2.75857e-76
Alpha-Beta	0.6	Accuracy	RMSD	-0.477962	2.55281e-120	-0.518858	4.37809e-145
All	All	Entropy	RMSD	0.160827	9.66338e-94	0.217371	1.82749e-171
Mainly Alpha	0.2	Entropy	RMSD	0.695966	1.71973e-58	0.715236	3.97986e-63
Mainly Beta	0.2	Entropy	RMSD	0.0724957	0.000145837	0.0963986	4.29356e-07
Alpha-Beta	0.2	Entropy	RMSD	0.572235	6.59832e-174	0.608614	1.69009e-202
Mainly Alpha	1.0	Entropy	RMSD	0.32269	3.8102e-11	0.35924	1.25209e-13
Mainly Beta	1.0	Entropy	RMSD	0.295059	1.18885e-58	0.367026	4.53285e-92
Alpha-Beta	1.0	Entropy	RMSD	0.473319	1.79585e-112	0.499859	3.2139e-127
Mainly Alpha	0.6	Entropy	RMSD	0.728486	2.2837e-67	0.782364	7.31436e-84
Mainly Beta	0.6	Entropy	RMSD	0.257023	5.62798e-45	0.287796	2.00541e-56
Alpha-Beta	0.6	Entropy	RMSD	0.475365	7.39946e-119	0.532351	5.02197e-154
All	All	Packing Density	Accuracy	0.501944	0	0.53743	0
Mainly Alpha	0.2	Packing Density	Accuracy	-0.0509211	0.312742	0.137834	0.00607275
Mainly Beta	0.2	Packing Density	Accuracy	0.0292804	0.125445	0.164092	5.42147e-18
Alpha-Beta	0.2	Packing Density	Accuracy	0.379528	2.29439e-69	0.372049	1.59287e-66
Mainly Alpha	1.0	Packing Density	Accuracy	0.395744	1.89666e-16	0.416918	2.98189e-18
Mainly Beta	1.0	Packing Density	Accuracy	0.258082	8.08204e-45	0.292361	1.43691e-57
Alpha-Beta	1.0	Packing Density	Accuracy	0.515746	1.08638e-136	0.516685	2.88705e-137
Mainly Alpha	0.6	Packing Density	Accuracy	0.647685	6.11114e-49	0.552489	2.40781e-33
Mainly Beta	0.6	Packing Density	Accuracy	0.098282	1.13785e-07	0.147498	1.425e-15
Alpha-Beta	0.6	Packing Density	Accuracy	0.402867	8.98781e-83	0.399854	1.86396e-81
All	All	Packing Density	Entropy	-0.0196157	0.0128103	-0.185332	2.24292e-124
Mainly Alpha	0.2	Packing Density	Entropy	-0.477704	6.55064e-24	-0.587934	4.34216e-38
Mainly Beta	0.2	Packing Density	Entropy	0.240969	1.70861e-37	-0.008196	0.668046
Alpha-Beta	0.2	Packing Density	Entropy	-0.45292	1.72486e-101	-0.505157	1.10878e-129
Mainly Alpha	1.0	Packing Density	Entropy	-0.261395	1.13269e-07	-0.252345	3.15235e-07
Mainly Beta	1.0	Packing Density	Entropy	-0.0666522	0.000356959	-0.19001	1.07187e-24
Alpha-Beta	1.0	Packing Density	Entropy	-0.496613	2.40773e-125	-0.500975	7.21699e-128
Mainly Alpha	0.6	Packing Density	Entropy	-0.80347	1.13261e-91	-0.787361	1.24796e-85
Mainly Beta	0.6	Packing Density	Entropy	0.0943589	3.57023e-07	-0.0658145	0.000390329
Alpha-Beta	0.6	Packing Density	Entropy	-0.445769	4.63934e-103	-0.546392	9.06595e-164
All	All	Packing Density	RMSD	-0.733224	0	-0.713059	0
Mainly Alpha	0.2	Packing Density	RMSD	-0.138153	0.00595513	-0.252268	3.76985e-07
Mainly Beta	0.2	Packing Density	RMSD	-0.317524	3.12801e-65	-0.519443	3.17717e-189
Alpha-Beta	0.2	Packing Density	RMSD	-0.276956	1.85825e-36	-0.371696	2.15964e-66
Mainly Alpha	1.0	Packing Density	RMSD	-0.662131	8.17734e-52	-0.682603	3.59499e-56
Mainly Beta	1.0	Packing Density	RMSD	-0.649463	0	-0.642015	0
Alpha-Beta	1.0	Packing Density	RMSD	-0.7393	0	-0.755926	0
Mainly Alpha	0.6	Packing Density	RMSD	-0.745023	5.50654e-72	-0.744231	9.33188e-72
Mainly Beta	0.6	Packing Density	RMSD	-0.651817	0	-0.65408	0
Alpha-Beta	0.6	Packing Density	RMSD	-0.674763	4.32909e-279	-0.464115	1.14316e-112
Mainly Alpha	0.2	Entropy	AlphaFold lDDT	-0.688717	7.67484e-57	-0.571755	1.15385e-35
Mainly Beta	0.2	Entropy	AlphaFold lDDT	-0.119032	4.10154e-10	-0.157457	1.13151e-16
Alpha-Beta	0.2	Entropy	AlphaFold lDDT	-0.483338	2.62108e-117	-0.530606	2.69442e-145
Mainly Alpha	1.0	Entropy	AlphaFold lDDT	-0.350669	5.12163e-13	-0.233948	2.24383e-06
Mainly Beta	1.0	Entropy	AlphaFold lDDT	-0.22921	1.81663e-35	-0.284929	1.19817e-54
Alpha-Beta	1.0	Entropy	AlphaFold lDDT	-0.535688	2.59553e-149	-0.484368	1.8733e-118
Mainly Alpha	0.6	Entropy	AlphaFold lDDT	-0.772877	1.24697e-80	-0.724328	2.92692e-66
Mainly Beta	0.6	Entropy	AlphaFold lDDT	-0.221967	1.06427e-33	-0.267678	9.00821e-49
Alpha-Beta	0.6	Entropy	AlphaFold lDDT	-0.481614	2.13787e-122	-0.500194	2.3041e-133

Table 2: Correlation Coefficients between sequence metrics and shape metrics of Monte-Carlo-sampled sequences.
